# Under Pressure: Response Urgency Modulates Striatal and Insula Activity during Decision-Making under Risk

**DOI:** 10.1371/journal.pone.0020942

**Published:** 2011-06-06

**Authors:** Catherine L. Jones, Ludovico Minati, Neil A. Harrison, Jamie Ward, Hugo D. Critchley

**Affiliations:** 1 Department of Psychiatry, Clinical Imaging Sciences Centre, Brighton and Sussex Medical School, Falmer, United Kingdom; 2 Department of Psychology, University of Sussex, Falmer, United Kingdom; 3 Department of Science, Fondazione IRCCS Istituto Neurologico “Carlo Besta”, Milano, Italy; 4 Sussex Partnership NHS Foundation Trust, Brighton, United Kingdom; 5 Sackler Centre for Consciousness Science, University of Sussex, Falmer, United Kingdom; Kyushu University, Graduate School of Systems Life Sciences, Japan

## Abstract

When deciding whether to bet in situations that involve potential monetary loss or gain (mixed gambles), a subjective sense of pressure can influence the evaluation of the expected utility associated with each choice option. Here, we explored how gambling decisions, their psychophysiological and neural counterparts are modulated by an induced sense of urgency to respond. Urgency influenced decision times and evoked heart rate responses, interacting with the expected value of each gamble. Using functional MRI, we observed that this interaction was associated with changes in the activity of the striatum, a critical region for both reward and choice selection, and within the insula, a region implicated as the substrate of affective feelings arising from interoceptive signals which influence motivational behavior. Our findings bridge current psychophysiological and neurobiological models of value representation and action-programming, identifying the striatum and insular cortex as the key substrates of decision-making under risk and urgency.

## Introduction

Making risky decisions under time pressure can lead to unfavorable consequences: “*Act in haste, repent at leisure*”. This effect is so well known that many countries even protect consumers against it with specific ‘cooling off period’ legislation. How does the brain choose under pressure? To date, little is known about the neural substrates which determine the effects of response pressure (or urgency) on decision-making behavior.

In financial decision-making paradigms, multiple components of each gamble are integrated in order to arrive at an optimal decision. The expected value (EV) of a choice option is defined as the sum of the value of each possible outcome weighted by each associated probability of occurrence. Rationally, options with a higher EV are favored, when all else is equal. However, EV maximization is not sufficient to account for all behavioural phenomena which are observed, such as risk aversion (where a sure payment is preferred to a risky option having equal or higher EV) and time discounting (where the subjective value of a payment decreases non-linearly with the associated delay). Influential models in behavioural economics including expected utility theory [1] and prospect theory [2] account for neuropsychological evidence [3], highlighting the integration of cognitive appraisal with emotional responses when making decisions under uncertainty.

Emotional reactions are typically faster than cognitive evaluations [4] and may constitute “affect heuristics” which reflect a speed-accuracy tradeoff during action-selection. Specific behavioural effects are observed when urgency is modulated by means of response time limitations. Information processing speed is increased [5], risky options are avoided if EV is positive and more frequently accepted if EV is negative [5], [6], and greater weight is placed on the negative domain, e.g. on potential loss [7]. On the basis of these studies, one can hypothesize that response urgency modulates more than one aspect of choice option evaluation, leading to heterogeneous effects on overall performance, depending on the specific paradigm.

Converging neuroimaging evidence indicates that EV and risk are computed in a distributed circuit, involving regions also supporting emotional processes. These include the striatum, the ventrolateral prefrontal cortex (vlPFC), the parietal cortices, the anterior cingulate and the amygdala [8] and more recently the insula [9]. Striatal activity is hypothesized to encode EV determined from the integration of multiple gamble parameters [10]; further, the striatum is the main candidate region implementing the speed-accuracy tradeoff and putatively acts as an “urgency switch” [11] to maintain a balance between decisional speed and accuracy [12], [13]. To date, it remains unclear to what extent these two aspects of striatal involvement in decision-making are anatomically overlapping and functionally integrated.

The striatum receives cortical projections from the insular cortex, which is emerging as a central hub in models of decision-making. Here, information concerning potential risk and reward is integrated with interoceptive (bodily) signals and the representation of time to give rise to momentary conscious experiences and “gut feelings” that strongly influence option selection [14], [15]. Clinical reports show that when the insula is damaged, addictive behavior is attenuated [16]. Patients with insula lesions also display relative insensitivity to EV [17], and impaired adjustment of subjective beliefs about risk through experience [18]. We therefore hypothesized that physiological and affective signals related to urgency may be represented in the insula, and functionally integrated with information concerning EV and/or uncertainty to modulate decision-making behavior.

**Figure 1:**
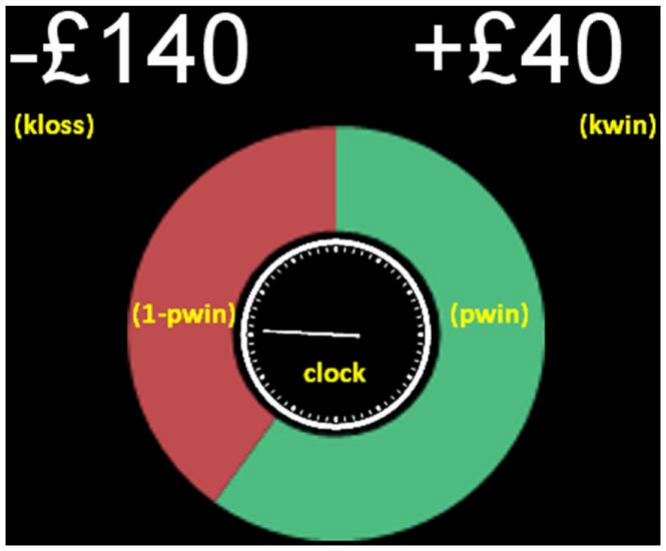
Example of gamble presentation. *kwin* represents potential gain, *pwin* the probability of winning, *kloss* potential loss. Participants were instructed to consider these elements together and equally weight them when deciding whether to bet or leave each gamble. A third of the gambles had positive EV (risk-advantageous), a third negative EV (risk-disadvantageous) and the remaining EV≈0 (risk-indifferent). The clock spun at two different rates: slow or fast. Participants were explicitly instructed that they always had 5 s to respond. Trials were interspersed with null events (fixation cross).

In this study, we investigated the behavioral consequences of subjective urgency on decision-making under risk. We used concurrent heart rate monitoring and functional magnetic resonance imaging (fMRI) to elucidate the psychophysiological correlates and neurobiological bases of this behavioral bias. In our task, participants were presented with mixed-gambles (see Fig. 1, below) involving an explicit probability of winning, a potential loss and a potential win, and chose whether to accept each gamble or avoid the risk. As cognitive processing is influenced by outcome feedback [19]–[21], we did not provide outcome feedback on a trial by trial basis, aiming to study the separate evaluation of each gamble, to probe the neural correlates of choice option evaluation and of the resulting internal competitive processes in the absence of learning and feedback-related emotional states.

Across trials, we varied EV and, in an orthogonal manner, the uncertainty associated with each gamble, through the probability of winning (*pwin*), the amount of money to be won (*kwin*) and the potential loss (*kloss*). This resulted in three levels of EV (defined as *pwin* × *kwin* - (1-*pwin*) × *kloss*): negative EV (risk-disadvantageous), neutral EV (risk-neutral) and positive EV (risk-advantageous gambles). We also defined two levels of uncertainty: probability class 20%/80% (low outcome uncertainty) and probability class 40%/60% (high outcome uncertainty). We characterized outcome uncertainty as being largest near *pwin*  = 50% [22], where the probability of receiving a reward or a loss are similar. EV and uncertainty were orthogonalized to test for distinct substrates involved in processing these parameters, and explore how urgency may differentially modulate their activity.

Our third factor, urgency, comprised two levels: high and low. On each trial, a fast or slow spinning clock was displayed along with the gamble to manipulate the subjective sense of urgency to make a response. Participants nevertheless always had a time limit of five seconds to respond on every trial (see Methods section), and were aware that this was identical irrespective of the clock speed.

We predicted that participants would predominantly accept gambles with a positive EV, reject those with negative EV and exhibit risk-aversion on gambles where EV≈0. We also predicted that a higher level of urgency would reduce decision time [23] and, through interaction with EV, would reduce performance accuracy. Specifically we anticipated that under high urgency participants would place fewer bets on positive EV gambles and bet more on negative EV gambles. Emotions consist of both subjective and physiological elements, and therefore, physiological responses during task performance provide insight into emotional processes [24], [25]. We predicted that manipulations of EV, uncertainty and urgency would modulate the participant's affective state in a manner that could be indexed by stimulus-evoked heart rate changes. At a neural level, we expected activation related to EV and/or uncertainty to be modulated by urgency state in regions implicated in action-selection, particularly the striatum and the insula.

## Results

### Behavioral responses

#### Effects of EV (positive, neutral, negative)

**Figure 2:**
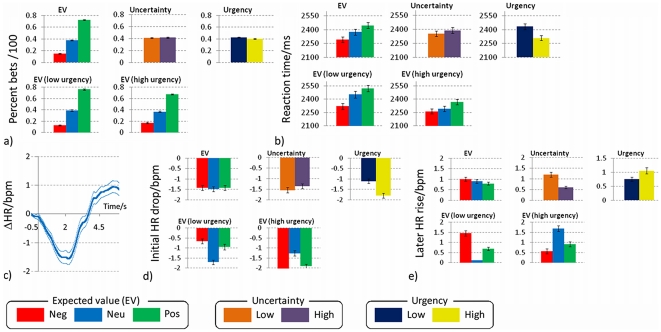
Behavioural and physiological results. Main effects and interactions of EV, uncertainty and urgency on percent bets (a) and reaction time (b). c) Average heart rate change (bpm) over single trials, dashed lines represent standard error of the mean. Main effects and interactions of EV, uncertainty and urgency on heart rate (HR) deceleration(d) and later rise (e).

As anticipated, EV significantly influenced the percentage of trials on which participants gambled (main effect of EV F_(2,26)_ = 115.8, p<0.001, η^2^ = 0.90), with significant differences between all EV levels (mean±SEM, positive EV: 72%±2%, neutral EV: 38%±2%, negative EV: 15%±3%, all post-hoc contrasts p<0.001) (Fig. 2a). There was also a main effect of EV on response time (F_(2,26)_ = 4.8, p = 0.02, η^2^ = 0.27) which was driven by significantly faster responses on negative EV trials (2290±30 ms) compared to neutral EV (2370±30 ms) and positive EV (2450±30 ms) trials (Fig. 2b). Performance level, defined as the ratio of rejected negative EV and taken positive EV gambles over the total number of negative EV and positive EV gambles, was 78±1% (range 60% to 91%) across participants. Risk propensity, defined as the proportion of neutral-EV gambles taken, was 38±1% across participants. A one sample t-test revealed that the group was significantly risk averse on neutral EV trials (i.e., participants tended to reject risk-indifferent gambles, t(13) = 2.9, p<0.01).

#### Effects of urgency (low, high) and its interaction with EV

There was a main effect of urgency on response times (F_(1,13)_ = 14.7 p<0.01, η^2^ = 0.53), with quicker responses for high-urgency trials (2310±30 ms vs. 2430±30 ms). There was also a significant interaction between urgency and EV (F_(2,26)_ = 3.7, p<0.05, η^2^ = 0.22) . Post-hoc contrasts showed that the effect of urgency was significantly more pronounced for neutral EV trials (2300±30 ms for high urgency vs. 2450±40 ms for low urgency) compared to negative EV trials (2260±30 ms for high urgency vs. 2320±30 ms for low urgency), (F_(2,26)_ = 9.9, p<0.01, η^2^ = 0.43) (Fig. 2b). There was also a statistical trend between urgency and EV on percentage bets (F_(2,26)_ = 3.0, p = 0.05). Post-hoc contrasts revealed that urgency differentially modulated the percent of bets on negative and positive gambles (F_(2,26)_ = 13.7, p<0.01): high urgency increased the proportion of negative EV gambles accepted (17±1% for high urgency vs.13±1% for low urgency) and decreased the proportion of positive EV accepted (68±1% for high urgency vs. 76±1% for low urgency) (Fig. 2a). Participants were not significantly more risk averse under high compared to low urgency (37% vs. 39% on neutral gambles).

#### Effects of uncertainty

There were no main effects of uncertainty on percent bets or response time and no interactions with EV or urgency.

#### Time estimation

To explore whether the rate of the spinning clock influenced the subjective perception of time, participants were asked to estimate short intervals under high (rapidly spinning clock) and low (slowly spinning clock) urgency conditions. This test was performed in the scanner immediately after completion of the gambling task. Participants overestimated the time elapsed under high urgency and underestimated it under low urgency (10±2% vs. −5±2%, F(1,13) = 20.5, p<0.001, η_p_^2^ = 0.59).

### Physiological responses

Gamble presentation elicited a biphasic orienting response in heart rate, consisting of an initial slowing followed by acceleration. Averaging over all participants, the initial bradycardic response peaked at −1.4±2.5 bpm, 1.8 s post-stimulus, with the subsequent acceleration peaking at 0.9±2.1 bpm, 5.4 s post-stimulus (Fig. 2c).

For the initial slowing (1.5–2.1 s post-stimulus), there was a main effect of urgency (F_(1,13)_ = 10.9 p = 0.006, η_p_^2^ = 0.46), with greater deceleration under high urgency (−1.8±0.4 bpm vs. −1.1±0.3 bpm). Further, urgency interacted with EV (F_(2,26)_ = 8.8, p<0.001, η_p_^2^ = 0.40), modulating heart rate deceleration for negative compared to neutral EV trials (p<0.001) and for positive compared to neutral EV trials (p<0.05). Under low urgency, neutral-EV gambles were associated with the greatest slowing, conversely under high urgency, negative-EV and positive-EV gambles evoked the greatest slowing compared to neutral-EV gambles (Fig. 2d)

For the later heart rate acceleration (5.1–5.7 s post-stimulus), there were no main effects of urgency, EV or uncertainty, however there was an interaction between EV and urgency (F_(2,26)_ = 6.1, p<0.01, η_p_^2^ = 0.32). Contrasts showed that urgency significantly modulated the difference in heart rate rise for negative compared to neutral EV trials (p<0.05) and for positive compared to neutral EV trials (p<0.05) (but not for negative versus positive, p = 0.3). Under low urgency, the greatest increase in heart rate was with negative EV gambles compared to high urgency where neutral EV gambles were associated with the greatest increase in heart rate (Fig. 2e).

To summarize behavioural and physiological results, urgency had a main effect on reaction times (high urgency elicited faster response times) and heart rate (high urgency elicited initial greater slowing) and significantly interacted with EV to modulate both measures. EV had a main effect on the number of bets taken and the response time. Uncertainty had no significant effects suggesting that the probability of gain/loss has little effect on behavior per-se, beyond its contribution in the definition of EV.

### Neuroimaging results

Throughout this section, only significant findings are explicitly reported. Those interactions that did not reach statistical significance threshold have not been reported.

#### Effects of EV (positive, neutral, negative)

**Figure 3:**
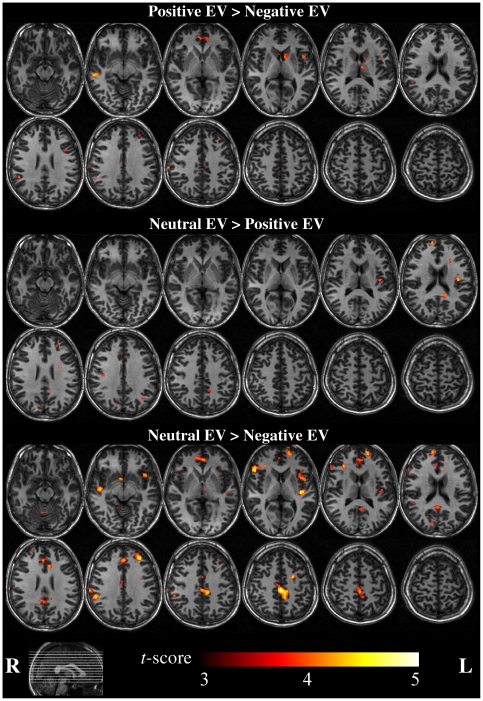
Whole-brain fMRI results for the main effect of EV. Activations shown at the cluster-forming threshold p<0.001. See Table 1 for full list of activation clusters and corresponding extents.

As predicted, positive-EV gambles (compared to negative EV) elicited greater activation within the striatum (head of the caudate bordering on the ventral striatum) reflecting greater reward expectation in this condition. Neutral-EV gambles elicited greater activation than both negative-EV and positive EV gambles bilaterally in the medial prefrontal and anterior cingulate cortex; the contrast with negative EV was associated with widespread differences, including in the dorsal- and ventrolateral prefrontal cortex and cingulate gyrus. Negative EV gambles did not elicit greater activation than positive- or neutral-EV gambles in any region (Fig. 3 and Table 1).

**Table 1:** Main effects of EV (whole brain analysis).

k_E_	Peak	MNI coords.	Side	Structure
**Positive EV > Negative EV**				
56	4.2	(58, −30, −10)	R	Middle temporal gyrus
40	3.9	(8, 14, 0)	R	Caudate nucleus
44	3.8	(54, −44, 28)	R	Supramarginal gyrus
35	3.7	(8, 46, −2)	R	Medial PFC, Anterior cingulate cyrus
72	3.7	(−8, 6, 6)	L	Caudate nucleus, Ventral striatum
	3.4	(−14, 8, −4)	L	
**Neutral EV > Positive EV**				
88	4.4	(−40, −16, 16)	L	Posterior insula
43	4.4	(14, −78, 28)	R	Cuneus
53	3.9	(12, 58, 16)	R	Medial PFC, Anterior cingulate cyrus
38	3.8	(−12, −48, 18)	L	Lingual gyrus
**Neutral EV > Negative EV**				
619	5	(2, −28, 44)	R	Posterior cingulate gyrus
	4.7	(−10, −30, 42)	L	
	4.2	(−5, −42, 48)	L	
133	4.7	(−16, 56, 8)	L	Medial PFC
	3.6	(−14, 68, 4)	L	
120	4.7	(56, −40, 34)	R	Supramarginal gyrus
192	4.6	(−32, 36, 32)	L	Dorsolateral PFC
	3.3	(−32, 52, 28)	L	
73	4.6	(32, 34, 8)	R	Ventrolateral PFC
183	4.5	(−40, −20, 4)	L	Posterior insula
232	4.4	(54, 24, 2)	R	Ventrolateral PFC
	3.6	(58, 32, 8)	R	
	3.4	(60, 24, 18)	R	
72	4.4	(12, 60, 16)	R	Medial PFC
88	4.3	(48, −16, −12)	R	Middle temporal gyrus
54	4.2	(−24, 0, 46)	L	Superior frontal gyrus
439	4.2	(2, 32, 30)	R	Anterior cingulate gyrus
	3.9	(−2, 44, −4)	L	
	3.8	(12, 48, −6)	R	
72	4.1	(2, −60, −24)	R	Cerebellum
	3.6	(4, −60, −32)	R	
	3.3	(12, −52, −14)	R	
66	4.0	(−10, 22, 22)	L	Anterior cingulate gyrus
186	4.0	(−42, 14, −12)	L	Anterior insula
	4.0	(−46, 6, 4)	L	
	3.6	(−38, 16, 0)	L	
63	4.0	(68, −28, 30)	R	Supramarginal gyrus
49	3.9	(4, −30, −30)	R	Pons
38	3.6	(26, 50, 22)	R	Dorsolateral PFC
	3.5	(20, 54, 28)	R	
163	3.6	(−2, −54, 12)	L	Posterior cingulate gyrus
	3.6	(10, −48, 24)	R	
	3.6	(2, −52, 22)	R	

No significant effects were observed for the contrasts not reported in this table. The parameter *k_E_* represents the number of 2×2×2 mm voxels in the cluster.

#### Effects of urgency (high, low) and interaction of urgency and EV

**Figure 4:**
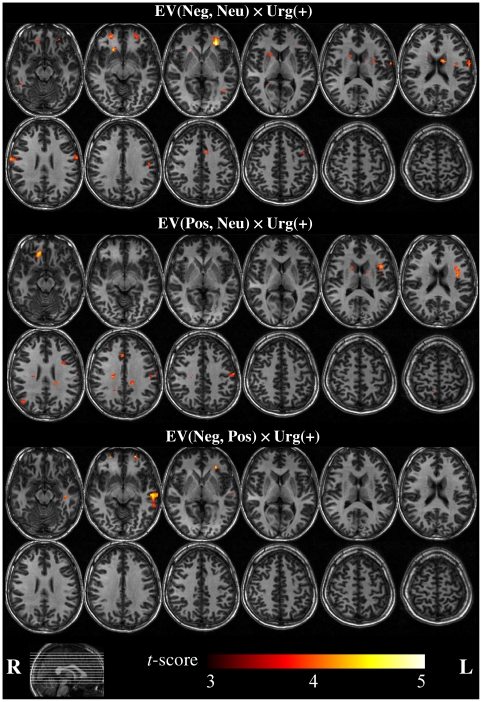
Whole-brain fMRI results for the interactions between EV and urgency. Activations shown at the cluster-forming threshold p<0.001. See Table 2 for full list of activation clusters and corresponding extents.

Critical to the main question of this study was the observation that urgency interacted with EV in many regions (Fig. 4 and Table 2). In particular, significant effects were observed for three contrasts. First, the difference between negative and neutral EV gambles was modulated by urgency for clusters in the ventrolateral and dorsolateral prefrontal cortex, striatum and insula: under low urgency, the response was greater for negative than neutral EV gambles, whereas under high urgency the effect was reversed. Second, the difference between positive and neutral EV gambles was modulated by urgency in the orbitofrontal cortex, anterior insula, cingulate gyrus, dorsolateral prefrontal cortex and caudate nucleus: under low urgency, the response was greater for positive than neutral EV gambles, whereas the converse was observed under high urgency. Third, the difference between negative and positive EV gambles in the middle temporal gyrus was modulated by urgency, i.e. the response was larger for negative than positive EV gambles under low urgency, and the effect was reversed under high urgency.

**Table 2:** Interactions between EV and urgency (whole brain analysis).

k_E_	Peak	MNI coords.	Side	Structure
**EV(Neg, Neu) × Urg(+)**				
208	5.4	(−28, 34, −4)	L	Ventrolateral PFC, Anterior insula
	3.7	(−26, 48, −8)	L	
	3.4	(−28, 42, −18)	L	
52	4.3	(20, 22, −10)	R	Ventral striatum
74	4.1	(−10, 0, 16)	L	Caudate nucleus
112	4.1	(44, −48, −24)	R	Cerebellum
	3.5	(40, −56, −28)	R	
79	4.0	(68, −6, 26)	R	Dorsolateral PFC
	3.6	(56, −10, 30)	R	
37	4.0	(−34, −8, 16)	L	Posterior insula
110	3.9	(−64, −6, 20)	L	Dorsolateral PFC
	3.7	(−64, −6, 10)	L	
42	3.8	(24, 50, −8)	R	Orbitofrontal cortex
46	3.8	(26, −50, 0)	R	Lingual gyrus
	3.8	(32, −56, 0)	R	
43	3.6	(18, 8, 8)	R	Caudate nucleus
	3.3	(24, 16, 4)	R	
**EV(Pos, Neu) × Urg(+)**				
82	4.3	(14, 38, −18)	R	Orbitofrontal cortex
222	4.1	(−42, 10, 10)	L	Anterior insula
	4.0	(−38, −6, 16)	L	
	3.8	(−38, 4, 16)	L	
38	3.9	(6, 22, 32)	R	Cingulate gyrus
77	3.8	(26, −20, 30)	R	
41	3.8	(−14, −2, 14)	L	Caudate nucleus
56	3.8	(−18, −34, 28)	L	
80	3.8	(−54, −18, 36)	L	Dorsolateral PFC
	3.7	(−62, −12, 26)	L	
	3.7	(−62, −16, 42)	L	
36	3.5	(18, 8, 10)	R	Caudate nucleus
	3.3	(12, 2, 8)	R	
**EV(Neg, Pos) × Urg(+)**				
180	4.5	(58, −24, −12)	R	Middle temporal gyrus
	3.9	(−68, −20, −10)	L	
	3.6	(−62, −44, −12)	L	

No significant effects were observed for the contrasts not reported in this table. The parameter *k_E_* represents the number of 2×2×2 mm voxels in the cluster. The interactions expanded are: i) EV(Neg,Neu)×Urg(+) = [EV(Neu)Urg(Hi)-EV(Neg)Urg (Hi)]-[EV(Neu)Urg (Lo)-EV(Neg)Urg (Lo)], ii) EV(Pos,Neu)×Urg(+) = [EV(Neu)Urg(Hi)-EV(Pos)Urg(Hi)]-[EV(Neu)Urg (Lo)-EV(Pos)Urg (Lo)] and iii) EV(Neg,Pos)×Urg(+) = [EV(Neg)Urg(Hi)-EV(Pos)Urg (Hi)-[EV(Neg)Urg(Lo)-EV(Pos)Urg (Lo)].

**Figure 5:**
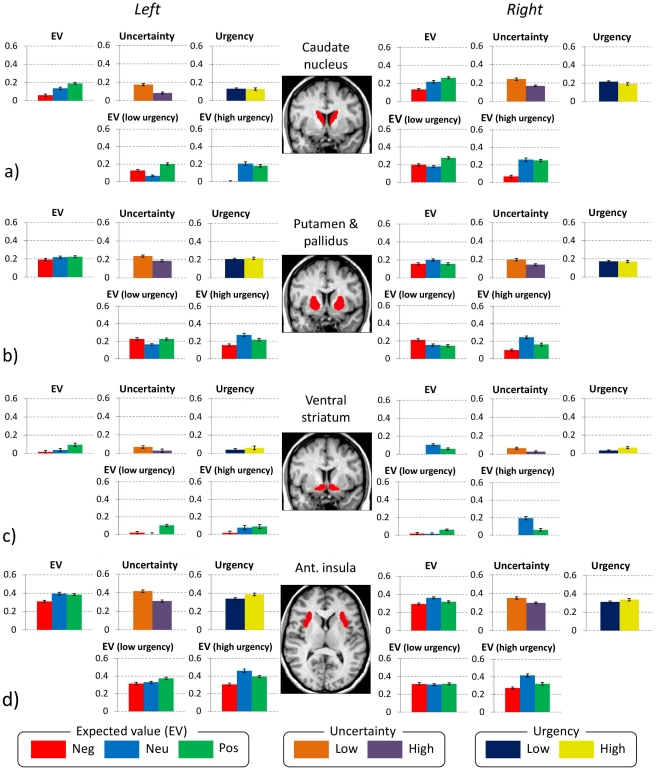
Region of interest plots for caudate nucleus, ventral striatum, putamen & pallidus and anterior insula. See text for statistical results.

To corroborate the above findings, region-of-interest (ROI) analyses were conducted on average activity in the caudate, ventral striatum, putamen and anterior insula (Fig. 5). For the caudate, there was a main effect of EV (F_(2,26)_ = 4.8, p<0.05, η_p_^2^ = 0.27) with stronger activation (BOLD signal percent change) for positive than negative EV (0.29±0.04% vs. 0.14±0.05%, p<0.05); neutral EV elicited activation of intermediate intensity (0.20±0.05%), without significant differences. Further, EV interacted with urgency (F_(2,26)_ = 4.6, p<0.05, η_p_^2^ = 0.26): post-hoc ANOVAs revealed that the effect of EV was stronger under high (F_(2,26)_ = 7.8, p<0.01, η_p_^2^ = 0.36) than under low urgency (F_(2,26)_ = 3.6, p<0.05, η_p_^2^ = 0.20). In particular, the response to negative EV gambles was significantly attenuated under high urgency (0.15±0.01% vs. 0.05±0.01%, p<0.02). There was no significant lateralization of any effect.

Within the putamen there were no interactions between EV and urgency, and no interactions of EV or urgency with side.

Within ventral striatum, there was a statistical trend towards a main effect of EV (F_(2,26)_ = 3.2, p = 0.05, η_p_^2^ = 0.19) which interacted with side (F_(2,26)_ = 4.7, p<0.05, η_p_^2^ = 0.25). Post-hoc ANOVAs revealed that the effect of EV was significant for the left (F_(2,26)_ = 6.4, p<0.01, η_p_^2^ = 0.31) but not for the right hemisphere (p = 0.2). There was no interaction between EV and urgency.

There was a main effect of EV in the anterior insula (F_(2,26)_ = 4.7, p<0.05, η_p_^2^ = 0.27), with neutral EV gambles eliciting the greatest activation. However, there were no interactions of EV, urgency or side.

#### Effects of uncertainty [low (20/80), high (40/60)]

At the whole-brain level, low-uncertainty gambles elicited diffusely stronger activation than high-uncertainty gambles bilaterally across regions including the insular cortex, the inferior and middle frontal gyri, anterior and posterior cingulate cortices, fusiform gyrus, angular gyrus, cuneus / precuneus and within the rostral brainstem and midbrain. The converse pattern was not observed in any region.

At the ROI level within the caudate, there was also a significant main effect of uncertainty (F_(1,13)_ = 7.2, p<0.05, η_p_^2^ = 0.4), with stronger activation for low uncertainty (0.21±0.05% vs. 0.13±0.04%). In the anterior insula there was a main effect of uncertainty (F_(1,13)_ = 15.8, p = 0.001, η_p_^2^ = 0.53), with stronger activation for low uncertainty (0.38±0.05% vs. 0.31±0.04%), and an interaction with side (F_(1,13)_ = 8.6, p<0.05, η_p_^2^ = 0.38). Post-hoc ANOVAs revealed that the effect of uncertainty was greater within the left (F_(1,13)_ = 23.3, p<0.001, η_p_^2^ = 0.62) than right hemisphere (F_(1,13)_ = 5.8, p<0.05, η_p_^2^ = 0.29). We did not observe any significant interactions between uncertainty and urgency and between uncertainty and EV in either the whole brain or ROI analysis.

## Discussion

Our results characterize how response urgency influences gambling behavior, and which brain regions subserve the observed behavioural effect. We successfully manipulated participants' urgency to make a decision to bet or not bet on mixed gambles, reflected in a significant reduction in response times on high urgency trials and corresponding changes in heart-rate. Corroborating data demonstrated that participants' subjective perception of time was altered when responding, in a time estimation task, where participants significantly underestimated the elapsed time in the high urgency condition and overestimated the elapsed time in the low urgency condition. Therefore an altered perception of the amount of time available to make a decision contributed to the reduced decision time under high urgency.

Participants predictably avoided loss by gambling less frequently on risk-disadvantageous trials (negative EV) and anticipated gain by gambling more frequently on risk advantageous trials (positive EV). EV also influenced response times, with participants making quicker decisions on gambles with greater potential loss (negative EV). These observations support the findings of Tom and colleagues (2007) who report faster response times to negative EV gambles, indicating a preference to attend to negative stimuli and a greater sensitivity to losses than gains [19], [26].

Critically, urgency interacted with the EV of a gamble to modulate behavior, heart rate and neural activity. At the behavioural level, high urgency was detrimental to performance, reducing the number of bets placed on risk advantageous (positive-EV) gambles and increasing the number of bets made on risk disadvantageous (negative-EV) gambles. It is suggested that the striatum may mediate this behavioural effect by releasing motor circuit inhibition to facilitate fast but possibly premature responses [12]. We provide empirical validation for this proposal by observing that increased urgency modulates activity within regions of the striatum that encode EV. Our results endorse a model of brain mechanisms for action-selection proposed by Redgrave and colleagues (1999) which suggests that, in order to be considered as a candidate substrate for action-selection, a neural system should exhibit certain properties, namely: 1) it should receive information about internal states and external cues relevant to decision-making, 2) it should support a mechanism to compute the level of urgency to be assigned to each available action, 3) it should have the capacity to resolve conflicts between competing actions based on their relative salience and 4) its outputs should be modifiable allowing the expression of winning actions while suppressing losing actions (importantly, this does not necessarily embed the requirement of a ‘linear’ relationship between activation level and EV, and indeed the relationship was more complex in our results). In this context, the striatum represents the putative core of a decision-making / action-selection circuit [27]. As discussed below, this circuit becomes engaged in a manner that depends on value not only in a ‘direct’ way, but also in terms of deviation from a situation of ‘risk-indifference’: In other words, engagement of action-selection circuitry is augmented under situations of high response conflict, as occurs in neutral-EV gambles.

The insula receives convergent information associated with salient environmental stimuli across sensory modalities, and direct modulation from the striatum [28], providing crucial capacity for incentive and hedonic signaling. We showed that urgency interacted with EV to modulate activity within insula (according to the whole-brain analysis) as well as modulating physiological arousal. While we are unable to demonstrate a direct relationship between physiological responses and behavioural choice, evidence from previous studies shows that somatic changes contribute to decision-making behavior [29]–[31] and can relate to how well an individual performs on a decision-making task [32]. Existing research on heart rate responses to emotional stimuli identifies two main markers: An initial bradycardia, thought to express attentional orienting to motivationally-salient events through parasympathetic activity [33] and a later rise in heart rate, signifying emotional arousal through sympathetic activation [34]. Greater initial decelerations are associated with more aversive stimuli [35], i.e. reflecting a “freeze” response. In the present study, higher urgency trials were associated with a more pronounced cardiac deceleration, 0–2 s following gamble presentation (prior to a response), signaling a more aversive emotional state (compared to low urgency trials). Since feedback and learning were absent in this task, we suggest that such physiological changes reflect affective components of the evaluation process itself, rather than anticipatory changes or reactions to reward or punishment. Urgency interacted with expected value to modulate both the initial heart rate deceleration and the later acceleration. The relationship between the orienting response (heart rate deceleration), neural activation and behavioural choice in decision-making is unlikely to be straightforward. The pattern of EV x urgency interaction observed on heart rate deceleration was not observed in the neuroimaging data, which indicate that, here, the regional neural activity observed as BOLD effects does not directly mediate the orienting response. The pattern of EV x urgency interaction observed on heart rate acceleration resembled more closely the interaction observed in the insula, i.e. greater heart rate increase for negative EV gambles under low urgency compared to high urgency trials, for which neutral EV gambles were associated with the largest heart rate increase. This suggests that insula activity is coupled to the sympathetic arousal generated by gamble evaluation.

Previous studies illustrate direct associations between physiological fluctuations (e.g. heart rate or skin conductance response) and activity changes within the insula [36]–[38]. Our study therefore highlights the contribution to emotional decision-making of brain regions known to represent these homeostatic states and, by implication, of those states themselves. Recent work relates the degree to which the insula is engaged during risky decisions that follow decisions involving no risk, to individual differences in urgency / impulsivity traits [39]. However, in this study the notion of ‘urgency’ differs categorically from that of the present study (e.g., as a state varying within individuals depending on external pressures versus a trait varying between individuals) as do other aspects of the experimental design (e.g. presence of outcome feedback). Current models of insula function also highlight the anterior insula as a key region involved in time perception [40]. How this information is integrated in emotional decision-making is becoming clearer, for example the insula and striatum are conjointly active in immediate versus delayed rewards [41] further supporting their role integrating temporal information with other parameters during decision-making under risk. In addition to insula and striatum involvement, dlPFC and vlPFC activation was observed in relation to the interaction between EV and urgency. The vlPFC processes motivational and emotional signals from subcortical areas and computes the behavioural salience of external events [42]; it is also involved in response inhibition [24]. In our data, the dlPFC and vlPFC responded to the contrast between neutral and negative EV, with enhanced activation for neural EV gambles. This effect was, in turn, modulated by the level of urgency in clusters in these regions. We hypothesize that the dlPFC and vlPFC, insula and striatum operate together as substrates for integrating affective information (e.g. related to urgency) into goal-directed behavior. A more general question for future research concerns how cortical and sub-cortical regions may differ in their facilitation of action programming.

Interestingly, in this study the insula and cingulate gyrus were most sensitive to gambles with a neutral value (EV≈0) and within the insula this effect was amplified under high urgency. An influential model of anterior cingulate function concerns its engagement in conflict monitoring, or “in situations requiring selection among a set of equally permissible responses”, a process particularly pertinent to the representation of neutral EV gambles. In particular, one can view decision-making as a competition between processes promoting and inhibiting the performance of a given action; in situations of high-conflict, internal competition leads to enhanced activity with respect to situations where one action rapidly wins over the other [43], [44]. One alternative account is that the conjoint activation of the insula and anterior cingulate to neutral EV gambles mediates risk aversion, as a subjective, emotionally driven phenomenon [45]. More specifically, we suggest that, depending on the level of urgency, the dominant neural representation shifts from an EV-centered one, where most regions respond linearly to EV, to a response conflict-centered one, whereby under high urgency the striatum, insula and cingulum are engaged proportionate to the level of deviation from a situation of risk-indifference (neutral vs. positive or negative EV).

Uncertainty was negatively correlated with activation in the insula cortex, the inferior frontal gyrus, the middle frontal gyrus, anterior and posterior cingulate cortices, fusiform gyrus, angular gyrus, cuneus/precuneus and within the rostral brainstem and midbrain. That is, we observed greater activation in these regions to gambles with probability class 20%/80% compared with 40%/60%. Neuroimaging studies have demonstrated that the probability with which a stimulus or response occurs modulates activity in the anterior insula, anterior and posterior cingulate cortex, middle frontal gyrus, ventromedial and dorsolateral prefrontal cortex [20], [37], [46]. Typically, these regions become engaged as the uncertainty associated with two possible outcomes increases. However, these studies do not dissociate the neural signals reflecting simple uncertainty of reward/loss from other decisional variables e.g. EV or reward/loss prediction errors following outcome presentation. Our findings therefore do not contradict these observations, because there are fundamental differences in task design and implementation across studies which are likely to account for distinct differences in patterns of activation, highlighting the complexity of uncertainty representation in the brain. In particular, one cannot exclude the existence of multiple neural representations underlying what appears to be a unitary definition of uncertainty, and these would be plausibly engaged in a highly task-dependent manner. Further, there may be effects related to shifts in cognitive strategy: When the probability of winning was close to 50%, especially under high urgency, participants might have approximated EV computation with a basic comparison between potential loss and gain, whereas when the probability of winning was distant from 50% deeper processing would be required to support choice option evaluation.

This study lays the foundations for further experiments detailing the effects of urgency on performance and associated physiological and neural substrates. This work could be extended to determine the relationship of physiological effects and/or neural activations with behavioural choice under different levels of urgency. This would involve considering how participants' choices (e.g. bet versus no bet) predicted their regional neural activation and/or heart rate change. The current experimental design did not permit us to assess this directly but illustrates the differential and integrative mechanisms associated with expected value, uncertainty and urgency on brain and body during the evaluation of the value of the options and associated risk. Furthermore, our experimental design did not enable dissection of sub-components of the urgency-inducing manipulation, for example effects of the spinning clock on emotional arousal and visual attention. Future work is necessary to qualify better effects of emotional versus attentional modulations on decision-making and the underlying neural activity. Beyond the mixed gamble design featured here, there is a potential need to examine effects of urgency separately within the loss and gain domains. The present paradigm is, however, well-suited for further exploration and future use in clinical and occupational populations, for example to study patients with gambling addiction or investment bankers, who likely exhibit different patterns of behavioural and physiological activity under heightened levels of urgency [47].

In conclusion, we provide evidence that signals related to response urgency are integrated with choice value information to modulate neural activity in the striatum and the insula, with concurrent changes in heart rate and gambling performance. This work informs current models of choice-selection, in which the striatum mediates fast but potentially inaccurate responses and supports the integration of basic decision-making parameters.

## Materials and Methods

### Participants

Fifteen right-handed, healthy participants (5 male, 10 female), mean age 25±10 yrs, were enrolled after written informed consent. The study was approved by the Brighton & Sussex University Hospitals NHS Trust ethics committee (Royal Sussex County Hospital, Eastern Road, Brighton, BN2 5BE, UK). All participants were free from neurological and psychiatric disorders.

### Design: gambling task

Sets of gambles were constructed based on three factors: EV, uncertainty (probability class) and urgency (Figure 1). For each gamble, EV and uncertainty were determined by independently manipulating the probability of winning (*pwin*), the amount of money to be won (*kwin*) and the amount of money that would be lost (*kloss*). This resulted in three levels of EV: negative, neutral and positive and two levels of uncertainty: high uncertainty (probability 40%/60%) and low uncertainty (probability 20%/80%). Urgency was defined as a two-level factor, and determined by the spinning rate of a clock presented alongside the gamble parameters. Participants chose whether to bet (i.e., to accept a risk related to the variable EV) or leave each gamble (thereby opting for a certain EV = 0 outcome). Participants were told that the outcome of each gamble was not pre-set, but would be determined at the time of response, and that the computer would track all earnings and losses in the form of a ‘virtual bank account’. Participants were instructed to respond within a maximum of 5 s, and that if they did not respond they would lose the fixed amount of £20.

A fast event-related fMRI design was employed, with an average inter-stimulus interval of 6 s and 9 presentations for each cell of the 2×2×3 experimental design, yielding a total of 108 experimental trials; 62 null-events of variable duration were also inserted. The total duration of the task was about 16 min. Stimuli were generated and presented using code developed in-house, based on the ‘Cogent’ toolbox (www.fil.ion.ucl.ac.uk/Cogent/). Stimulus timing and order were optimised by means of optseq2 (www.surfer.nmr.mgh.harvard.edu/optseq).

### Procedure

Participants were informed they would receive feedback on their performance at the end of the experiment. All participants were rewarded with £15 for their participation. Stimuli were presented on a projection screen, viewed through a mirror attached to the head coil. Prior to scanning, participants were shown some sample gambles to familiarize themselves with the stimuli and were encouraged to consider all three elements of each gamble, weighting them equally. Participants were naïve to the concept of EV, and were told not to attempt to perform any explicit calculation. Following completion of the gambling task, they performed a time estimation test to examine whether this was affected by the clock spinning rate. For this part, the same clock was displayed, without a gamble. Participants pressed a button when they thought that the given time interval had elapsed. The time interval to estimate, i.e. 1, 2 or 3 s, was indicated before each trial.

### Data Acquisition

Functional imaging was performed on a clinical 1.5 T scanner (Magnetom Avanto, Siemens AG, Erlangen, Germany) equipped with a standard 4-channel head coil. Sequential T2*-weighted echo-planar images were acquired with bi-commissural orientation for 21 slices, 5 mm thickness, no gap, TR = 2000 ms, TE = 50 ms, in-slice resolution 2×2 mm, matrix 80×128. Structural images were acquired with a magnetization-prepared rapid gradient-echo sequence, having 0.9 mm isotropic voxels and TR = 1160 ms, TE = 4.44 ms, FoV 230×230 mm, matrix size 256x256, 50 slices.

### Behavioral Data Analysis

For the gambling task a 3 (EV: positive, neutral, negative) by 2 (Urgency: high, low) by 2 (uncertainty: high, low) ANOVA was performed with percent bets and response time as dependent variables. For each participant, performance was calculated as the ratio of rejected negative EV and taken positive EV gambles to the total number of negative EV and positive EV gambles. We set a minimum accuracy threshold of 60%, leading to the rejection of one participant. Risk propensity was indexed by the average number of bets on neutral EV trials. A standard threshold of p<0.05 was assumed for significance of behavioural and psychophysiological data.

### Physiological analysis

Heart rate changes were assessed through an MRI-compatible pulse oximeter (Nonin 5400, Nonin Inc., Plymouth MN, USA). The plethysmographic signal was low-pass filtered at 1 Hz and processed with a peak-picking algorithm yielding beat-by-beat heart rate measurements. The resulting signal was epoched in the [−0.5,5] s peristimulus range and averaged across trials. On the basis of the grand average (see Fig. 2c), two measurement windows were defined: [1.5,2.1] s and [5.1,5.7] s. Resulting values were processed with a within-subject ANOVA as described above.

### Imaging data preprocessing and analysis

Functional imaging data were analyzed using SPM8 (Wellcome Centre for Neuroimaging, London UK). Functional scans were realigned and unwarped, slice-timing corrected, and co-registered with individual anatomy. Subsequently, all scans were transformed into standard Montreal Neurological Institute (MNI) stereotactic space and smoothed using an 8 mm FWHM Gaussian filter.

At the individual level, design matrices were set up using 12 separate regressors, accounting for each combination of EV (3 levels), urgency (2 levels), uncertainty (2 levels). Each event was modeled as having a duration equal to the measured response time. Movement parameters were also inserted as nuisance covariates. Statistical maps were generated for each of the 12 design conditions, contrasting activation for that condition with respect to null events. At the second level, these maps were entered as a 3×2×2 flexible factorial analysis, which enabled us to determine the main effects of EV, urgency and uncertainty, as well as their interactions. We set a voxel-level threshold of p<0.001, uncorrected, to form clusters. Subsequently we employed a Monte Carlo method, involving a full model of the acquisition, normalization and smoothing steps, to calculate the cluster extent threshold yielding an effective cluster-level α of 0.05, which was 35 voxels; this approach has specific advantages as it is parsimonious in terms of statistical assumptions and explicitly captures the effects of the data preprocessing steps [48].

### ROI analysis

Planned regions-of-interest (ROI) for the caudate nucleus, putamen, ventral striatum and anterior insula were adapted from the AAL atlas [49], by an experienced operator who separated the anterior/posterior insula and dorsal/ventral striatum drawing on the three planes on a canonical brain in normalized space. Average BOLD signal percent change was extracted, averaged for each ROI and analyzed by means of a 3 (EV) x 2 (Uncertainty) x 2 (Urgency) x 2 (side) repeated measures ANOVA. Bonferroni's correction for multiple comparisons was applied, accounting for multiple comparisons within each ROI.
